# Design of a Chlorophyll Fluorescence Sensor Head for Continuous On-Leaf Measurements

**DOI:** 10.1021/acsomega.6c03026

**Published:** 2026-06-25

**Authors:** Johannes Klueppel, Samaneh Baghbani, Stefan J. Rupitsch, Laura M. Comella

**Affiliations:** † Department of Microsystems Engineering, 9174 Albert-Ludwigs-Universität Freiburg, Georges-Koehler-Allee 102, 79110 Freiburg, Germany; ‡ Institute of Energy Efficient Mobility, Hochschule Karlsruhe Technik und Wirtschaft, Moltkestrasse 30, 76133 Karlsruhe, Germany

## Abstract

Continuous monitoring of physiological activity is increasingly important for environmental observation systems that track ecosystem responses to drought stressors. Chlorophyll fluorescence (ChlF) is a sensitive, early indicator of drought-induced stress in trees that is directly measured on leaves and needles. However, existing autonomous ChlF systems are typically bulky and disturb natural leaf movement or provide insufficient excitation intensity for reliable measurements. Here, we present a leaf-wearable sensor head specifically engineered for long-term, autonomous environmental monitoring in forests. The design integrates a high-intensity excitation interface based on a blue LED, delivering up to 9000 μmol m^–2^ s^–1^. The operation of the LED directly on the leaf enables energy-efficient excitation. To facilitate energy-aware design of field sensing systems, we introduce the metric photon density efficacy (μmol m^–2^ s^–1^ mW^–1^) for quantifying excitation efficiency in power-constrained fluorescence sensors. To ensure stable measurements under environmental forcing, the sensor head incorporates a 4.1 g lightweight Y-shaped magnetic attachment structure with a soft silicone interface, designed to maintain constant sensor–leaf geometry while allowing natural leaf motion. Mechanical characterization demonstrated a mean pull-off force of 3.41 N, while field tests confirmed reliable sensor operation under wind speeds up to 21.5 m s^–1^. The presented design enables the nonintrusive integration of optical sensors directly on leaves and needles for extended monitoring periods. This work establishes a new hardware approach for distributed leaf-level sensing within environmental monitoring networks, enabling high-resolution observation of vegetation physiological dynamics across spatial and temporal scales.

## Graphical abstract

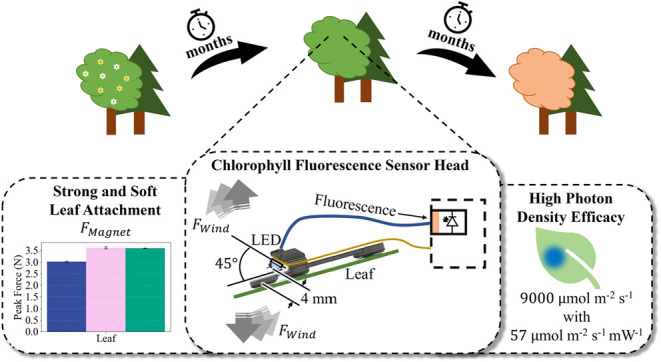

## Introduction

Forests play a significant role as part of the water cycle, in climate regulation, and as the dominant component of the land carbon sink.^1,2^ The rapid climate changes in recent years have brought increasing mean temperatures and more prolonged drought periods, which create physiological stress in trees.^3^ Especially, the unprecedented drought from 2018 to 2020 in central Europe led to high tree mortality.^1^ Established measurement approaches for forest observation are reaching their limits because they cannot reflect the sheer volume of changing processes and the incredible speed of those changes. To follow up, large measurement networks with high temporal and spatial resolution of measurements are required to deepen our understanding of stress reactions in forests and to develop mitigation strategies.^4^ An early indicator of drought-induced stress is chlorophyll fluorescence (ChlF).^5^ ChlF is light emitted from leaves and needles in the near-infrared (NIR) region following the absorption of an external light source as part of the photosynthesis process. ChlF competes with photochemistry and can serve as a proxy for the efficiency of photosynthesis in a leaf.^6^

Measuring optical parameters such as fluorescence, reflectance, and imaging-based assessments across large sensor networks requires novel technological solutions with high temporal and spatial resolution. In particular, sensor systems must remain attached directly to the leaf throughout the entire growing season. This requires extremely careful leaf handling, as continuous measurements on the same leaf are essential for generating consistent data sets. The sensors must combine strong yet soft adhesion that maintains attachment for several months without causing damage or detachment while remaining compact and lightweight so as not to restrict the leaf’s natural movement. Commercially autonomous systems for ChlF, such as the MICRO-PAM (Heinz Walz GmbH, Effeltrich, Germany), have significant weight and size, which requires fixing the leaf to the sensor system instead of vice versa, hindering the leaf’s natural movement.^7^ Based on our own experience, during strong wind events, the leaves are ripped from the MICRO-PAM sensor head and are sometimes destroyed. Existing ChlF sensor designs in the literature are unsuitable for attaching to leaves due to their size and weight.^8–11^ Lightweight and flexible solutions exist for sensors that measure the reflectance of plant tissue, but they continuously cover the measurement area.^12,13^ Further plant-wearable sensor systems, that attach directly to leaves, measure strain or gas levels.^14,15^ These wearable sensors typically comprise a soft, flexible substrate. However, they exhibit similar drawbacks, such as affecting leaf physiology by covering the measurement area and restricting light transmission and air permeability.^13,15,16^ Furthermore, recent review papers highlight that most studies do not demonstrate adequate stability and durability under harsh environmental conditions.^14,15^ To obtain reliable on-leaf ChlF measurements, we need to face an unaffected and uncovered leaf area at a constant angle and distance for several weeks. Hence, no reliable, leaf-attachable sensor interface for optical parameters is currently available either commercially or in the scientific literature. Additionally, access to tree crowns typically relies on climbers, cranes, or towers, making the deployment and maintenance of numerous sensor units both cost- and labor-intensive. Consequently, we need sensor systems that require minimal maintenance. Therefore, for the goal of long-term deployment, the sensors must operate autonomously with ultralow power consumption and energy-efficient designs that will be supported by energy harvesting in the future.

In this work, we propose the mechanical design of a plant wearable sensor head for long-term ChlF measurement campaigns. The focus of this paper is the energy-efficient and high-power sensor interface, including its reliable, noninvasive attachment concept that maintains stable positioning over the entire growing period. Side-by-side ChlF measurements against those of commercial systems are outside the scope of this work. A detailed evaluation of the fluorescence measurement performance is provided in ref,^17^ where the plant-wearable sensor system described here is used as the sensor head for the readout electronics.

In our work, we address energy-efficient long-term deployment through careful design of the optical components. In particular, the selection of the excitation light source is critical for low-power operation. The key parameters we consider are the type of light source, its emission wavelength, and the required optical intensity. Moreover, we quantify our light source with a newly introduced photon density efficacy metric tailored for a better comparison of ChlF sensor systems regarding the energy required for the excitation of ChlF. In addition, the opto-mechanical design plays a central role in ensuring reliable measurements. Therefore, we analyze and compare different light-guiding concepts for our system in terms of source–detector distance and collection angle to ensure consistent and reliable sensor data. Furthermore, we address the challenge of long-term deployment without damaging the leaves. We evaluate several approaches for achieving both strong and soft attachment of sensors to plant leaves over an entire growing season. Our design choices are guided by the need for environmental robustness and a compact, lightweight, and preferably transparent form factor in order to minimize both the impact on the leaf and any influence on the measurement results. We rely on commercially available materials and components to facilitate small-series production. To validate the sensor head for use in forest conditions, we first quantify the attachment strength through tensile testing and then demonstrate the reliability of the attachment during a field deployment lasting several weeks. Our work aids researchers in building more energy-efficient and autonomous optical sensors. With the proposed leaf attachment, this work paves the way toward a long-term wearable sensor head capable of hosting different types of sensors on a leaf for several months without affecting its natural behavior, with ChlF sensing serving as one example application.

## System Design

### Chlorophyll Fluorescence Sensor Interface

Fluorescence sensor interfaces, in general, consist of an excitation light source, an excitation filter, an emission filter, and an emission detector.^18^ The careful choice of each component is essential for an energy-efficient ChlF sensor to achieve a high signal-to-noise ratio in the most efficient way since ChlF accounts for only about 1–2% of the total absorbed light.^6^

#### Excitation Light Source

The most common method used to measure stress using ChlF is pulse amplitude modulation (PAM).^19^ In PAM, two types of pulses with different intensities are utilized. The intensity of the excitation light is measured as the number of photons in μmol m^–2^ s^–1^. The first pulse type uses microsecond pulsed measuring light (ML) to excite modulated fluorescence within a low intensity range of 0.0065 μmol m^–2^ s^–1^ to 1.5 μmol m^–2^ s^–1^. The second pulse type is applied to saturate the reaction centers in the photosystem II of leaves with too many photons with a much more intense saturation pulse (SP).^20^ Based on our experience, sunlight can reach intensities of more than 2000 μmol m^–2^ s^–1^ in the absorption range during summer at noon. To reliably saturate the leaves under high light conditions, the light source should be capable of exceeding the natural sunlight intensity by multiple times. Commercial devices, therefore, provide saturation pulses of 4000 to 8000 μmol m^–2^ s^–1^.^20^ Depending on the plant species and physiological state, even higher intensities may be required. In this work, ChlF refers to the fluorescence of chlorophyll **a**.

For an efficient ChlF excitation, a light source with a wavelength close to one of the two absorption peaks of chlorophyll **a** around 430 and 665 nm should be chosen.^21^ To utilize most of the light and to reach the high required intensities efficiently, a high optical directivity of the light source is beneficial. Based on these requirements, two types of light sources can be considered for embedding in a small sensor: LEDs and laser diodes. LEDs are very common in commercial devices, such as the MICRO-PAM, which uses a blue power LED having a maximum emission at 465 nm, and the MINI-PAM-II, which uses a red 655 nm LED (both devices: Heinz Walz GmbH, Effeltrich, Germany). Additionally, in the literature, blue LEDs are used for ChlF excitation.^8,9,11^ LEDs have the advantage of high availability in different versions of wavelengths, sizes, and power. Furthermore, they exhibit a good power conversion efficiency (PCE) of up to 93% for blue LEDs.^22^ In order to achieve high optical directivity, additional lens assemblies are required, which affect the overall package size. Alternatively, laser diodes have been utilized to excite ChlF, offering coherent light emission and thereby eliminating the need for an excitation filter.^10,23^ Lasers also provide high directivity, thereby reducing omnidirectional dispersion. However, conventional laser diodes, such as those used in ref 10 are through-hole technology (THT) components with a diameter of 5.6 mm, which limits system miniaturization, increases weight, and raises costs compared to LED-based solutions. Furthermore, this laser diode is classified as a Class 3B laser, as it must exit the housing to excite ChlF. Class 3B lasers require special safety standards, such as protective eyewear, which makes the device unsuitable for use in public spaces like forests.

For the sensor design presented in this work, the LED was selected as the excitation source because it has wide availability in compact form factors and can be obtained with sufficiently narrow viewing angles. Our design objective is to maximize the number of photons delivered to the region of interest while minimizing the required electrical input power. Although the reported photon efficacy (photons J^–1^) of red LEDs exceeds that of blue LEDs,^22^ we selected blue LEDs as the excitation source for two reasons: (1) InGaN-based blue LEDs exhibit superior temperature stability,^24^ and (2) the shorter excitation wavelength increases the spectral separation between excitation light and fluorescence emission, thereby simplifying the optical filtering. The use of blue light can, depending on the specific spectra, enable the omission of a dedicated emission filter, which further reduces optical complexity and module size. In particular, the 465 nm blue LED 150141BS63130 (Würth Elektronik, Waldenburg, Germany) with a footprint of 2.8 mm × 3.5 mm is used in this work.

#### Opto-Mechanical Design

Commercial systems are often configured with the electronics in an external housing, using only an optical fiber to guide the excitation light to the leaf and to guide the resulting fluorescence back to the detector. To separate the excitation and emission light paths, a dichroic mirror in the housing is required. This configuration is commonly used in devices manufactured by WALZ. The second option is to place both the light source and detector close to the leaf with a direct optical path. This configuration is often found in the literature.^8,9^

The first configuration introduces optical losses when coupling light into the fiber and through splitting on the dichroic mirror. This is not desirable if the system is energy constrained. Therefore, to design an energy-efficient sensor head, it is beneficial to place the light source close to the sample with a direct optical path. In the second configuration, in addition to the light source, there is a detector with an amplification circuit made from discrete components placed close to the sample. This would introduce additional size and weight to the sensor head, which would hinder the leaves’ natural movement. In our application, we have chosen to combine both approaches. We attached the LED close to the leaf to create a short and direct optical path for the excitation light. The emitted fluorescence is guided by a 1.5-m long optical-grade plastic fiber (Edmund Optics, Barrington, NJ, USA) made of poly­(methylmethacrylat) (PMMA) with a diameter of 1.5 mm to the detector. At the end of the fiber, in front of the detector, there is a 6 mm × 6 mm × 2 mm long-pass filter (Edmund Optics, Barrington, NJ, USA) with a cut-on wavelength of 650 nm to separate the excitation and emission wavelengths. Both the LED and the optical fiber are oriented toward the leaf at an angle of 45° (see Figure 1a), with a common focal point located 4 mm from the surface between the attachment mechanism (see Figure 1b). We will discuss the attachment mechanism in the following sections. This geometry minimizes shadowing of the monitored area while maximizing the collection of fluorescence. To ensure that both angle and distance remain constant as the leaf moves, the components must be attached using a strong and rigid mechanical support.

**Figure 1:**
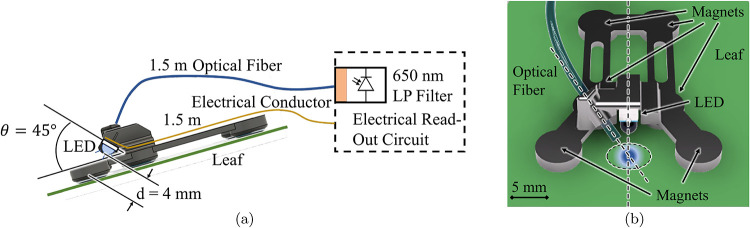
Opto-mechanical design with LED directly on the leaf to excite the fluorescence. (a) The optical fiber guides the emitted fluorescence from the leaf through a 650-nm long-pass (LP) filter to the electric read-out circuit. The design ensures a constant angle and distance of the optics to the leaf and enables consistent fluorescence excitation, even under movement. (b) This geometry enables a common, uncovered focal point located 4 mm from the surface, between the attachment mechanism.

### Photosynthetically Active Radiation Sensor

To calculate the electron transport rate (ETR) from the fluorescence measurements, the intensity of photosynthetically active radiation (PAR) is required. Therefore, a PAR sensor based on the AS7341 multispectral sensor (ams-OSRAM AG, Premstaetten, Austria) is embedded in the sensor head. It is an off-the-shelf product, and after proper calibration, this sensor offers accuracy comparable to that of commercial single-channel devices.^25^ The multispectral sensor is covered with a diffuser L-52 (Kimoto LTD, Cedartown, GA, USA) and clear resin to protect it from water and UV radiation. The PAR sensor must be mounted in such a way that it measures PAR perpendicular to the leaf surface, ensuring an accurate determination of the PAR incident on the ChlF sensing area.

### Temperature Sensor

The temperature of the leaf is measured from the underside with an SHT45 (Sensirion AG, Staefa, Switzerland) temperature sensor.^26^ The sensor must be pressed against the underside of the leaf to ensure direct contact between the leaf and the sensor.

### Transparent Substrate

Attaching sensors to leaves over extended periods requires a careful assessment of their impact on the leaf physiology. A healthy leaf performing photosynthesis requires sufficient light as an energy source, gas exchange with the surrounding air, and unhindered water transport and evaporation. These constraints favor a transparent design that minimizes direct contact with the leaf for both the carrier material and the substrate of the printed circuit boards (PCBs).^27^

The carrier material, which mechanically integrates the sensors, the optical fiber, and the leaf attachment mechanism, is made from the transparent Clear Resin V4 (Formlabs Inc., Somerville, MA, USA) to exploit the high design flexibility of additive manufacturing. The resulting structure is shown in Figure 1. It is crucial to ensure a stable and well-defined angle and distance between the sensor interfaces and the leaf surface to keep the sensor measurements consistent. The overall footprint is small, and the measuring area remains uncovered so that leaf evaporation is not significantly affected. The original 3D-design files from Figure 3a are available in the Supporting Information in popular file formats.^28^

To electrically connect the LED and the communication lines to the temperature and PAR sensor, a flexible PCB is required. Transparent, flexible PCB materials that are occasionally available commercially are poly­(ethylene terephthalate) (PET) or poly­(ethylene naphthalate) (PEN).^29^ However, it is noted that PET, especially under wet conditions, undergoes accelerated degradation due to UV light and is therefore unsuitable for outdoor use.^30^ Further PCB concepts are based on clear poly­(dimethylsiloxane) (PDMS). It demonstrates transparency and good UV stability.^31–33^ However, these processes are not yet industrially mature and are unsuitable for small-series production.

Flexible PCBs manufactured from opaque polyimide (PI) are well-established in the industry, have good environmental resistance, and can be ordered in any quantity with assembled components. Since the focus of this work is more on reliability and industrial manufacturing scale than on transparency, we opted for a tradeoff and used PI PCBs. The PCB in Figure 3a is designed as a narrow strip of 5 mm, and since it does not come into direct contact with the surface of the leaf, the shading influence is not an issue. The original PCB design files are available in the Supporting Information.^28^

### Leaf Attachment

With the ChlF sensor interface, additional sensors and substrates now defined, an appropriate attachment mechanism is required. Various approaches have been proposed for attaching sensors to leaves: a simple mechanical solution uses a clip mounted on white-covered FR4 material for up to six weeks, but this caused an undesired local increase in temperature at the attachment point.^34^ The next proposed solution, microhooks that penetrate the leaf tissue, offered good short-term adhesion but involved invasive connection to the leaf, with potential tissue damage and limited evidence for long-term use.^35^ In a less invasive approach, a perforated silicone membrane was used to enable adhesion between the sensor and the leaf. The membrane is flexible and allows light, gas, and water vapor to pass, but the adhesion may be negatively affected by rain, which could limit reliability under field conditions.^36^ Some works attached precured Ecoflex directly to the leaf, but this method is only intended for single use.^13^ Similarly, sensor patches that are attached with medical tape to the leaf also shade the underlying leaf area, which risks altering the local microclimate and physiological processes.^16^ Another proposed solution was the use of silicone-coated magnets. On the one hand, this design caused shadowing beneath the magnets, which led to the discoloration of the shaded regions of the leaf. On the other hand, it has been reported to allow long-term deployment and to withstand high wind speeds without causing mechanical damage to the leaf itself.^37^

Our work aims to attach a sensor head to leaves for several months while ensuring resistance to wind and rain without harming the leaf or influencing physiological processes. For this purpose, the magnetic solution seems to be the most promising, as it combines mechanical stability with soft attachment through silicone pads. We adapted the proposed solution for the ChlF sensor, using six pairs of Neodymium disk magnets, each with a 4 mm diameter and 1 mm height, glued to the carrier. We expect a physiological response in the leaf area directly beneath the silicone-covered magnets due to local shading. Previous studies reported a reduction in chlorophyll content under similar attachments for a gas-measuring cuvette^37^ and pressure measurements.^38^ We consider this response an accepted tradeoff since it does not influence the measuring area.

The procedure used to embed and encapsulate the magnets in the 3D-printed attachment is illustrated in Figure 2. Each side of the transparent attachment has six cavities for the magnets. The cavities are located under the circular shapes of the substrate visible in Figures 1 and 3. First, a thin, uniform layer of 2-component epoxy adhesive (UHU GmbH, Buehl, Germany) was applied to the inner surface of the magnet cavity (a). The magnet (*r* = 2 mm, *h* = 1 mm) was then placed into the cavity (b), ensuring the prescribed polarity: one orientation was used for the bottom part and the opposite orientation for the top part. The exposed magnet surface was subsequently cleaned with isopropanol and allowed to dry for 10–20 min (c). After drying, primer was applied to the cleaned surface (d) and left to react for the recommended time of 15–90 min (e). Ecoflex 00–20 (Smooth-On Inc., Macungie, PA, USA) was then cast over the magnet and its lateral regions until it formed a slight convex dome (f). The Ecoflex was cured for at least 2 days at room temperature (g). Finally, the cured Ecoflex was coated with Parylene C to provide protection against moisture (h).

**Figure 2:**
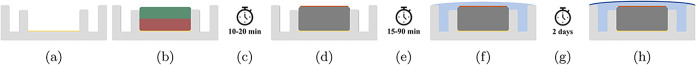
(a) First, glue is applied in the magnet cavity. (b) Then, the magnet is placed onto the glue, with one orientation for the bottom part and the opposite orientation for the top part. (c) The magnet surface is cleaned with isopropanol, and a waiting time of 10 to 20 min is observed. (d) Afterward, the primer is applied, and (e) another waiting period is observed. (f) Then, Ecoflex is cast on top, and the cavity is filled to the sides as well. (g) The Ecoflex is allowed to cure for a minimum of 2 days at room temperature. (h) In the end, the Ecoflex is coated with Parylene C to provide protection from moisture.

### Assembled Sensor Head

The assembled sensor head in Figure 3 consists of an optical fiber, a semitransparent top attachment with magnets, a flexible PCB carrying the temperature sensor, a blue LED, a multispectral PAR sensor, and a semitransparent bottom attachment with magnets (a). In the assembled configuration (b), the temperature sensor is glued to the bottom part of the attachment and is oriented toward the lower leaf surface. This setup ensures that only the small sensor tip touches the surface, allowing for maximum air circulation and preventing local heating effects. It follows a well-established practice in environmental and plant physiological measurements (e.g., Walz MICRO-PAM^39^). Thermocouples or similar point sensors are placed in direct contact with the leaf without significantly altering leaf temperature or photosynthesis. The LED and optical fiber are directed toward the upper leaf surface at an angle of 45° angle, and the PAR sensor faces upward. The flexible PCB and the top attachment are also glued together using 2-component epoxy adhesive. When attached to a leaf (c), the temperature sensor records the leaf temperature on the underside, ChlF is measured on the upper side via the optical fiber after excitation through the LED, and ambient light is recorded by the PAR sensor.

**Figure 3:**
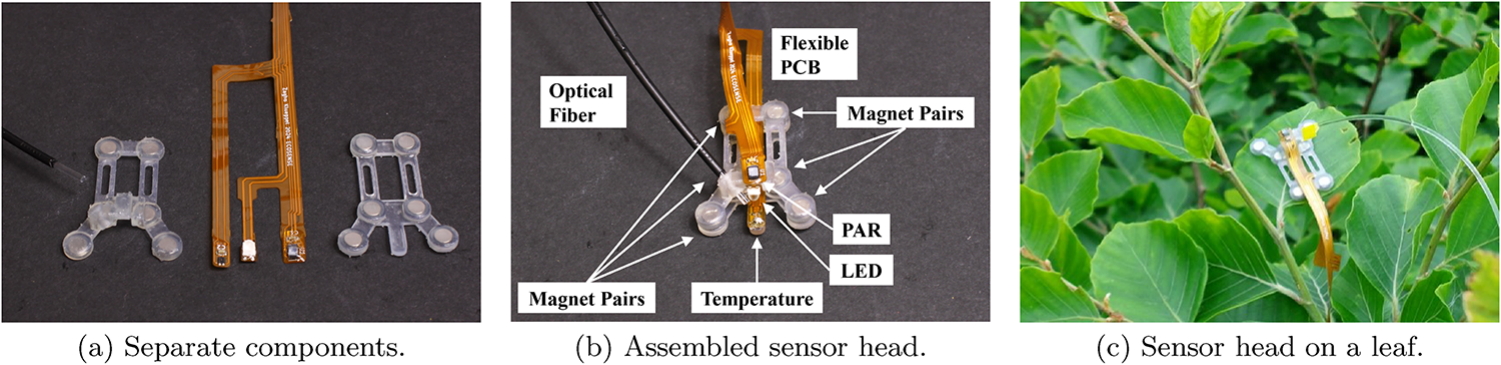
Final sensor head in different situations. (a) Optical fiber, top attachment, flexible PCB with temperature sensor, LED, and multispectral PAR sensor, and bottom attachment. (b) Assembled components with the temperature sensor upside down, LED and optical fiber pointing to the leaf, and the PAR sensor looking upward, measuring the ambient light. (c) Sensor head attached to a leaf, temperature sensor hidden, measuring on the underside of the leaf, ChlF at the upside, and ambient light orthogonal to the leaf surface. Photograph courtesy of Clara Stock. Copyright 2025.

The Y-shaped attachment spans the leaf like a canvas in front of the sensor interface and maintains a constant distance and angle, even during strong wind events. The constant distance is crucial to ensure consistent LED intensity at the leaf surface for every measurement. In addition, the Y-shaped design does not cover the measurement area, thereby preserving natural illumination conditions for unaffected photosynthesis. The design also ensures an orthogonal alignment of the PAR sensor relative to the leaf surface, enabling accurate measurements of the incident light on the leaf, independent of leaf angle or orientation. With a size of 26.7 mm × 35.5 mm × 11.7 mm and a weight of 4.1 g, this sensor head design allows for the natural movement of the leaf and makes it a true wearable for leaves. The 2.7 g and 1.5 m long optical fiber and the 1.5 m power and communication cable are wrapped around the branch and do not impose additional load on the leaf.

## Materials and Methods

### Photosynthetic Photon Flux Density

The PAR range is defined as the light from 400 to 700 nm that drives photosynthesis.^40^ One photon within the PAR range drives one reaction in the reaction center of the photosystem in leaves, independent of the wavelength. Consequently, in photosynthesis research, light intensity is typically quantified as the photon flux per unit surface area and per unit time instead of energy.^41^ This is called the photosynthetic photon flux density (PPFD) and is expressed in units of μmol m^–2^ s^–1^. To convert the energy of a monochromatic light source into the corresponding photon count, the following equation is applied

1 $$I^{Q}\left(\lambda_{p}\right)=\frac{\lambda_{p}\cdot I^{E}\left(\lambda_{p}\right)}{N_{A}\cdot h\cdot c}\cdot 10^{6}$$

The PPFD *I* ^Q^(λ_p_) is converted from the irradiance in energy units *I* ^E^(λ_p_) to the equivalent quantum unit with the peak wavelength λ_p_ of the monochromatic light source, Planck’s constant *h*, the speed of light *c*, and Avogadro’s number *N*_A_.

### LED Characterization

#### Total Power

The LED was positioned as close as possible in front of the S120C power sensor (Thorlabs, Newton, New Jersey, USA) to capture the total optical power *P*_opt,total_ of the beam. A Keithley SMU 2450 source meter (Tektronix, Beaverton, Oregon, USA) was utilized to power the LED, adjusting current levels incrementally from 1 to 5 mA in 1 mA steps and from 10 to 70 mA in 10 mA steps. The electrical power *P*_el,total_ was derived from the current settings and voltage measurements obtained via a multimeter. Measurements of both electrical and optical power were stored at each specified current level.

#### Irradiance

Since exciting ChlF requires a significant amount of photons, we used only the part of the light within the full width at half-maximum (FWHM) to calculate the projected area. For most of the LEDs, the viewing angle 2θ at FWHM is known from the datasheet. Depending on our experience, the given θ in the datasheet often does not reflect reality and provides only an orientation. Therefore, we measured the beam profile ourselves to obtain an accurate value using the self-built setup in Figure 4.

**Figure 4:**
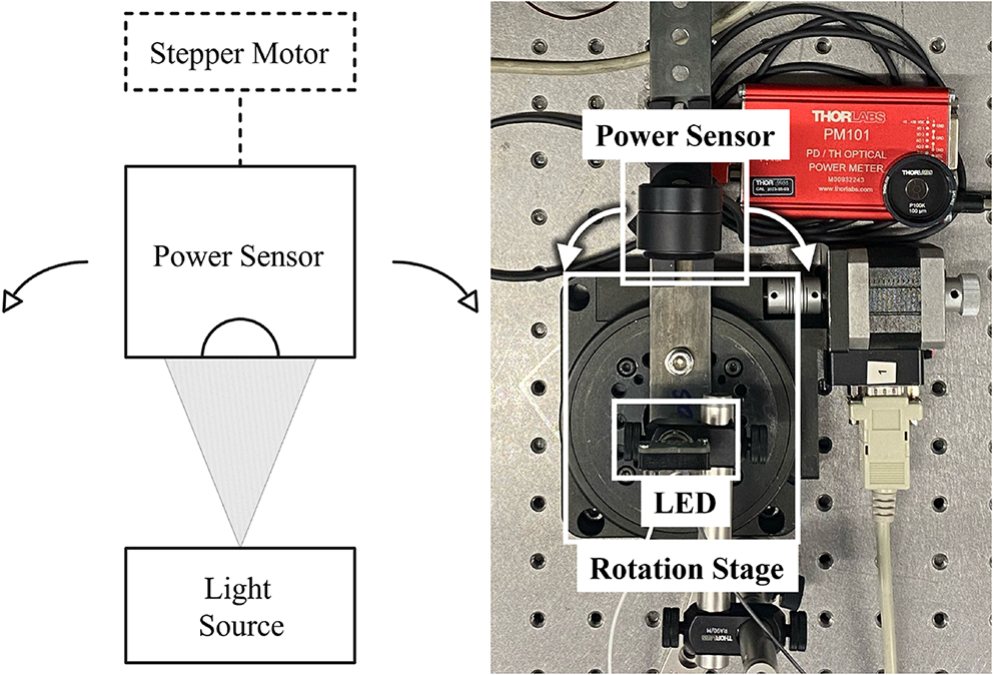
Setup to measure the viewing angle of the LED. The LED is positioned centrally above a rotation stage, while the power sensor is placed atop a rod, rotating around the LED. The beam intensity is measured at each degree from −25° to 25°. Photograph courtesy 6 of Nicolas Leclerc. Copyright 2025.

The LED was mounted centrally above a rotation stage and was driven at 30 mA. The power sensor was mounted on a rod, covered with a 100 μm pinhole and facing the LED at a distance of 10 cm. The rod was turned by the rotation stage around the LED. This method returns a spatial irradiance distribution *I* ^E^(θ). To obtain the FWHM radius *r*_FWHM_ of the projected beam at a distance *d*, the radius was calculated using the angle θ_FWHM_ at FWHM from the measurement in Figure 4

2 $$r_{\mathrm{FWHM}}=\tan\left(\theta_{\mathrm{FWHM}}\right)\cdot d+r_{\mathrm{offset}}$$

Since the LED is not a point source, we need to add an *r*_offset_, which is the radius of the LED lens, as an approximation. Because we were only interested in the power within the FWHM boundaries, the measured total optical power needs to be scaled to the area of the FWHM. The FWHM power ratio is calculated by dividing the integrated spatial irradiance distribution within the FWHM boundaries by the integrated total spatial irradiance distribution:

3 $$\eta_{\mathrm{FWHM}}=\frac{\int_{\theta_{l}}^{\theta_{r}}I^{E}\left(\theta \right)\theta d\theta }{\int_{-\inf}^{\inf}I^{E}\left(\theta \right)\theta d\theta }$$

With θ_*l*_ representing the angle of the left FWHM border and θ_*r*_ representing the angle of the right FWHM border. The beam’s total power was scaled to the FWHM area with the ratio η

4 $$P_{\mathrm{opt},\mathrm{FWHM}}=P_{\mathrm{opt},\mathrm{total}}\cdot \eta_{\mathrm{FWHM}}$$

The projected FWHM area was calculated using the corresponding radius *r*_FWHM_ calculated in eq 2

5 $$A_{\mathrm{FWHM}}=\pi \cdot r_{\mathrm{FWHM}}^{2}$$

The irradiance *I*_fwhm_ was calculated by dividing *P*_FWHM_ by the area *A*_FWHM_

6 $$I_{\mathrm{FWHM}}^{E}=\frac{P_{\mathrm{opt},\mathrm{FWHM}}}{A_{\mathrm{FWHM}}}$$

#### Photon Density Efficacy

We introduce photon density efficacy as a metric that quantifies how efficiently a light source converts electrical power into photons that are actually available for fluorescence excitation within the leaf. In contrast to established metrics in the literature, which are defined from the emitter’s perspective and characterize only the ability of an LED to convert electrical energy into photons per time unit (μmol s^–1^ mW^–1^), photon density efficacy explicitly accounts for the photons per time unit and area that reach and interact with the leaf tissue.^22^

We extend the definition to the receiver’s perspective and include the area to the metric: μmol m^–2^ s^–1^ mW^–1^

7 $$\eta_{\mathrm{ph}}=\frac{I_{\mathrm{FWHM}}^{Q}}{P_{\mathrm{el}}}$$

The quantum unit *I*_FWHM_^Q^ is converted from the energy unit irradiance *I*_FWHM_^E^ using eq 1. *P*_el_ is the electrical power, and η_ph_ is the efficacy parameter for the photon density. This photon density efficacy enables a better comparison of ChlF sensors in terms of energy efficiency. However, from eq 2, it is evident that this characterization is only valid under a constant distance *d*, which makes reliable leaf attachment unavoidable.

### Leaf Attachment

#### Pull Force Test

To characterize the force that holds the sensor head attached to the leaf, we executed a pull force test. We prepared three attachment samples according to the section Leaf Attachment. Each sample was tested across three attachment configurations to allow for better comparison: empty, paper, and leaf. For the empty configuration, nothing was placed between the attachment components, for paper, we used two sheets of normal paper (70 g m^–2^) with a total thickness of 170 μm, and for the leaf configuration, we used a 300-μm-thick evergreen ivy leaf. For each configuration, three replicate tests were conducted, resulting in a total of nine measurements per sample. Figure 5 demonstrates the paper configuration in the left image before the peak force. The tensile testing machine pulled the attachment apart until it reached a displacement of 5 mm. In the right image of Figure 5, the two halves of the attachment are separated after reaching the peak force. This setup enabled the quantification of attachment strength and the comparison of different configurations. Mean values and standard deviations were calculated across replicates to assess the reproducibility and reliability of the magnet attachment. In the Supporting Information, we provide a video of the pull test.^28^

**Figure 5:**
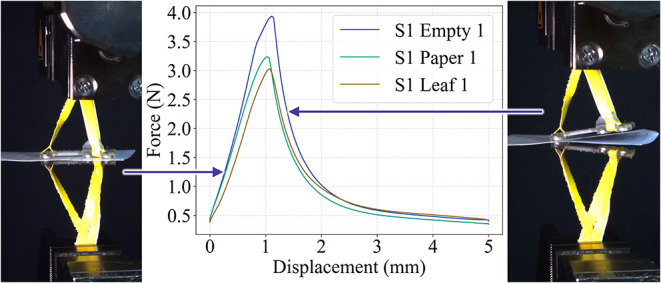
Screenshots of the pull test. The clamping devices of the tensile testing machine can be seen at the top and bottom of both images, visible in a metallic color. The leaf attachment is fixed with tape stripes between the clamps. The configuration with 170 μm paper between the top and bottom magnetic attachment is visible in the images. The left image was captured on the force–displacement curve before the peak force was reached, whereas the right image was taken after the peak force had been reached and the attachment had separated.

#### Field Test

During the summer season of 2025, a total of 33 ChlF devices equipped with the newly designed sensor head were deployed at the ECOSENSE field site in the Black Forest, Germany, during different periods from July to November.^42^ A total of 16 sensor heads were attached to the leaves of a European beech (*Fagus sylvatica* L.), while 17 sensor heads were attached to the needles of a Douglas fir (*Pseudotsuga menziesii* MIRBEL). The first sensor heads were deployed in July, ensuring that the beech leaves had reached their full size. The objective of this field experiment was to evaluate the mechanical reliability of the leaf attachment mechanism, the flexible PCB, and the PMMA-based optical fiber under realistic environmental conditions. To facilitate parallel development within our research group and accelerate deployment for field testing, we are currently operating the ChlF devices using a 24 VDC power cable rather than solar energy harvesting. In parallel, our group is investigating the pathways toward fully autonomous operation. In previous studies, we investigated the energy budget, data transfer strategies, and electrical architecture required for a solar-powered, batteryless, energy harvesting sensor node.^43,44^ These developments are intended to support future autonomous versions of the sensor nodes.

Throughout the long-term deployment, the sensor heads were continuously exposed to natural weathering including UV radiation, variable humidity, and strong wind events. Meteorological data (e.g., relative humidity, wind speed, and global radiation) were recorded on site with a local weather station operated by our group, as described in ref 42. At the end of the deployment period, we assessed the number of surviving sensor heads and identified mechanical failure modes and design weaknesses based on visual inspection and functional testing of each device.

## Results and Discussion

### LED Characterization

#### Total Power

Table 1 shows the results for the LED at four different driving currents, ranging from 1 mA to 50 mA. In the column “Efficiency”, it is evident that the relationship between electrical and optical power is not constant. The highest efficiency of 32.9% was reached at 3 mA. With increasing current, the efficiency decreases to 21.9% for 50 mA. The reason for this behavior lies in the junction temperature of the LED, which rises with higher currents and leads to a reduction in efficiency.^24^

**Table 1:** Calculated PPFD and Efficacy at 4 mm Distance with an Illuminated FWHM Area of 11.3 mm^2^

current in mA	el. power in mW	opt. power in mW	efficiency in %	*I*_FWHM_^E^ in W m^–2^	PPFD_FWHM_ in μmol m^–2^ s^–1^	efficacy in μmol m^–2^ s^–1^ mW^–1^
1.0	2.48	0.59	31.5	52.38	203.47	82.04
3.0	7.62	1.88	32.9	168.11	635.04	85.70
10.0	26.70	6.24	31.2	557.95	2167.38	81.17
50.0	159.50	26.20	21.9	2341.65	9096.23	57.03

#### Irradiance

Figure 6 shows a cross-section of the LED beam and highlights its main angular characteristics. The measured beam exhibits a total FWHM viewing angle of 19.5° (from −8.8° to 10.7°), which was significantly narrower and more directive than the 30° specified in the datasheet. Using the FWHM limits in eq 3, the fraction of the total emitted power contained within the FWHM region was determined. The corresponding irradiated area was obtained from eq 5, with the beam radius derived from the measured angles in eq 2. Table 1 summarizes the resulting power and irradiance values for different drive currents at a fixed LED-to-target distance of 4 mm.

**Figure 6:**
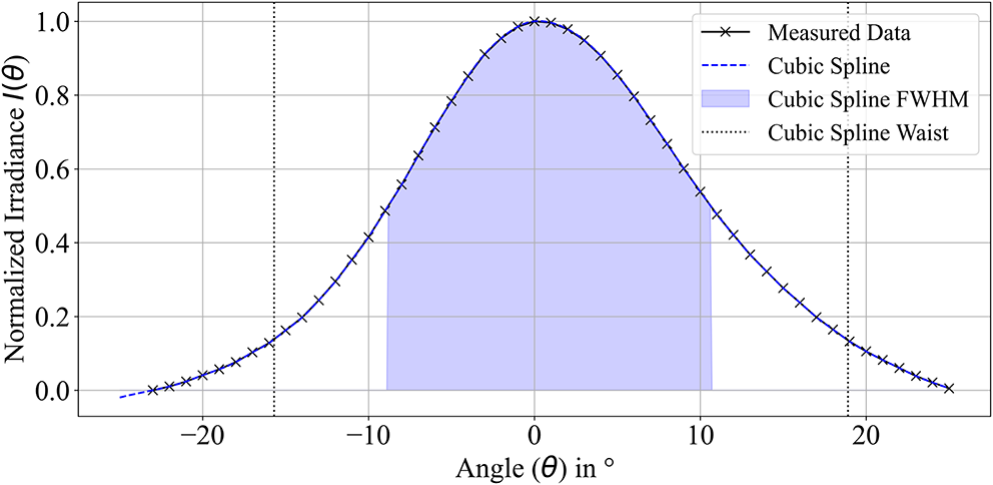
Normalized power of the LED plot over the radiance angle. The actual measured data in black crosses. Additionally, a cubic spline interpolation in blue.

#### Photosynthetic Photon Flux Density

The requirements for the ChlF excitation are given as 0.1 μmol m^–2^ s^–1^ for the measuring light to 8000 μmol m^–2^ s^–1^ for the saturation pulse. The PPFD of the LED in our design, with a distance of 4 mm from the LED tip to the leaf surface, is sufficient to cover the entire range. Table 1 shows the results of the PPFD calculated with eq 1. In Table 1, the PPFD is calculated for different driving currents of the LED. To obtain a sufficiently low measuring light, the LED is pulsed at 10 Hz with an on-time of 10 μs at higher currents. To saturate the ChlF, a minimum pulse duration of one second at 50 mA is required to achieve more than 8000 μmol m^–2^ s^–1^.

#### Photon Density Efficacy

Since the relationship between electrical and optical power was not constant for different currents, the photon density efficacy was also not constant. Therefore, each current in Table 1 has its own photon density efficacy value. The maximum efficacy of the LED is obtained at 85.70 μmol m^–2^ s^–1^ mW^–1^ for a driving current of 3 mA. To run the ChlF sensor in the most energy-efficient manner, the goal is to drive the LED for the fluorescence excitation close to this maximum. But higher light intensities in the ML, in combination with short pulses, improve the signal-to-noise ratio, while still maintaining a low average excitation light. Furthermore, to achieve a reliable saturation pulse, the LED drive current must be approximately 50 mA to reach a PPFD exceeding 8000 μmol m^–2^ s^–1^. Therefore, an operation close to the efficiency maximum is not possible. For energy-efficient operation, this intensity can be adjusted according to ambient light conditions measured by the PAR sensor on top of the sensor head: at lower ambient light levels, the required intensity can be reduced. This offers two benefits: (1) the overall power consumption decreases, and (2) the photon density efficacy of the system increases.

### Leaf Attachment

#### Pull Force Test

The pull test was used to evaluate the mechanical strength of the magnet attachments for the leaf sensor across three different configurations: empty, paper, and leaf. The highest mean peak force was observed until the first magnet lost its connection, as visible in the video in the Supporting Information (“video_force_*configuration*.wmv”).^28^ Since magnet strength is dependent on the distance between the magnets, to make a better comparison to the literature, we characterized our attachment in different configurations. For the empty configuration, the force reached 4.84 (±0.66) N. The configuration with a 170 μm paper showed a slightly lower mean peak force of 3.77 (±0.39) N, while the attachment with the 300 μm leaf produced the lowest mean peak force of 3.41 (±0.30) N. Figure 7 shows the results of the pull tests for all samples (S1, S2, and S3) in each configuration with error bars over the three repetitions.

**Figure 7:**
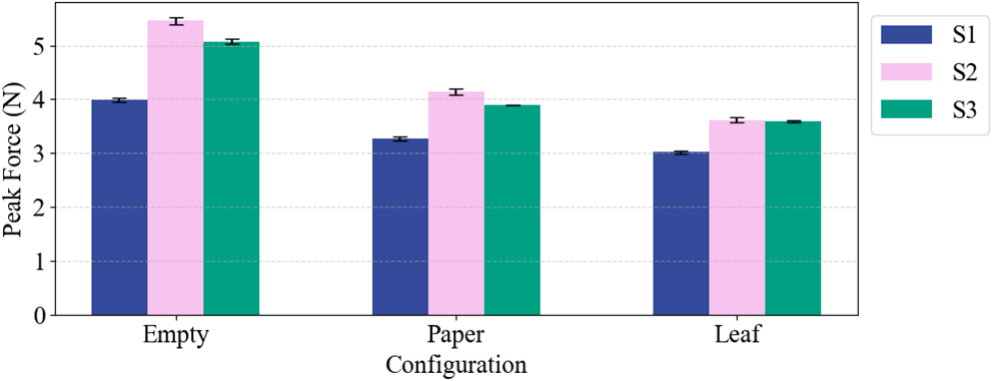
Peak forces of the pull test of all three samples with standard deviation over the three configurations.

In comparison to the values of other leaf attachment methods in Table 2, our method demonstrates the highest reported attachment force with minimal harm to the leaf.

**Table 2:** Overview of Various Attachment Methods and Their Associated Forces and Harm^a^

publication	attachment method	pull force	harm
Thalheimer et al.^34^	clip	–	buckle, cover, heat
Fiorello et al.^45^	hooks	up to 1.3 Ncm^–2^	penetration
Zhao et al.^36^	silbione RT gel	up to 0.3 N	–
Zhang et al.^13^	ecoflex adhesion	up to 5 Nm^1–^	–
Lu et al.^16^	medical tapes	–	–
Frey et al.^37^	magnet ring	–	discolouration
this work (empty)	6 magnet pairs	4.84 (±0.66) N	discolouration
this work (170 μm paper)	*r* = 2 mm, *h* = 1 mm	3.77 (±0.39) N	
this work (300 μm leaf)		3.41 (±0.30) N	

a Force values of this work are the mean average with standard deviation over the three samples.

#### Field Test

A total of 33 sensor heads were deployed in the forest. The longest deployment period was almost 18 weeks, and the mean deployment duration was nine weeks. Of these 33 sensor heads, 21 remained fully functional throughout their entire deployment. Eight experienced a broken optical fiber, three suffered a ripped-off flexible PCB, and one had a mechanically damaged sensor head, but the magnet attachment itself never failed. We attribute the observed failures primarily to mechanical stress occurring during strong wind events. The sensor heads were mounted in close proximity to the tower structure and to neighboring branches. As a result, the branches carrying the sensor heads experienced frequent collisions, both branch-to-branch and branch-to-tower, under repeated, dynamic movements with wind gusts of up to 21.5 m s^–1^. Furthermore, it is known that the mechanical strength of PMMA degrades under outdoor conditions, including intense UV radiation and high humidity, as evidenced by a reduced elastic modulus.^46,47^ These external influences lead to localized stress concentrations in the optical polymer fiber, as well as in the substrate of the flexible printed circuit board, making them more vulnerable to mechanical stress. A Supporting Video recorded during installation illustrates the strong wind gusts that typically occur in tree crowns (”video_node_in_forest_wind.mp4”).^28^

Overall, these findings underline the robustness and suitability of this sensor head design for use in the leaf sensor systems. We consider it a natural next step for the entire ChlF sensor system to find small and lightweight solutions for the integration of the readout electronics directly into the sensor head, making the optical fiber obsolete and increasing the mechanical stability. Other hazardous environmental factors, such as yellowing of the polymers due to UV radiation, could not be observed during the deployment periods. On the leaf side, no further harmful incidents, such as leaf ripping, have been recorded. Similar to refs,^37,38^ we observed discoloration beneath the magnets (see Table 2). The measuring areas showed no visual harm that could be transferred to reduced light (discoloration) or local hot spots (brown spots).

## Conclusion

In this work, we developed and validated an optical sensor head explicitly tailored for long-term on-leaf ChlF measurements. Our design philosophy was guided by two primary requirements for season-long deployment: (i) an energy-efficient design to enable fully autonomous operation and (ii) a minimally invasive, mechanically robust form factor that preserves the health and natural behavior of the leaves. To this end, we realized a compact, lightweight, and largely transparent sensor head based on industry-available components, paving the way for small series production and practical field deployment.

We introduced a Y-shaped leaf attachment that maintains a constant sensor–leaf distance, even under strong winds, while allowing the natural movement of the plant. Mechanical characterization demonstrated a mean pull-off force of 3.41 N when attached to a leaf, representing, to our knowledge, the strongest leaf attachment reported in the literature that remains harmless to the leaf tissue. On the opto-electronic side, we characterized the excitation light source with a newly proposed photon density efficacy metric that quantifies how efficiently a light source converts electrical power into photons that are actually available for fluorescence excitation in the leaf. This efficacy value enables a quantitative comparison of energy efficiency between different ChlF sensor designs and directly supports the development of future low-power, energy-constrained systems. Field tests with 33 deployed devices over several weeks demonstrated the feasibility of our approach under realistic forest conditions. Of these, 21 devices operated successfully over the test period, while 12 failed due to mechanical damage induced by wind, highlighting both the robustness of the concept and the remaining engineering challenges for extreme weather resilience. Two aspects remain for future work. First, to enhance mechanical stability, replacing the optical fiber with a miniaturized on-leaf readout circuit represents a logical next step. Second, the use of nontransparent PCBs may further reduce optical interference with the leaf.

Overall, the results support our claim that the presented multisensor probe is a credible step toward “deploy-and-forget” leaf wearables for long-term sensor networks in environmental and agricultural science. Its design enables reliable measurement of ChlF and with some adaptations of other optical parameters, such as reflectance or imaging-based assessments. With additional solar energy harvesting, these nodes will gain enough energy for battery-less, autonomous operation. Such networks will be key to resolving plant physiological dynamics across scales and seasons, ultimately improving our understanding of ecosystem responses to a changing climate and informing more sustainable management practices.

## Supplementary Material



## Notes

KiCAD production files for the flexible PCB; bill of materials for the flexible PCB; 3D printing files in popular file formats; videos of the pull force test; and a video of the sensor in the forest exposed to strong wind (ZIP)
